# Neurodevelopment and Metabolism in the Maternal-Placental-Fetal Unit

**DOI:** 10.1001/jamanetworkopen.2024.13399

**Published:** 2024-05-28

**Authors:** Mariana Parenti, Rebecca J. Schmidt, Daniel J. Tancredi, Irva Hertz-Picciotto, Cheryl K. Walker, Carolyn M. Slupsky

**Affiliations:** 1 Department of Nutrition, University of California, Davis; 2 Now with Center for Developmental Biology and Regenerative Medicine, Seattle Children’s Research Institute, Seattle, Washington; 3 Department of Public Health Sciences, University of California, Davis; 4 MIND Institute, University of California, Davis, Sacramento; 5 Department of Pediatrics, School of Medicine, University of California, Davis; 6 Department of Obstetrics & Gynecology, School of Medicine, University of California, Davis, Sacramento; 7 Department of Food Science and Technology, University of California, Davis

## Abstract

**Importance:**

Disturbances in maternal, placental, and fetal metabolism are associated with developmental outcomes. Associations of maternal, placental, and fetal metabolism with subsequent neurodevelopmental outcomes in the child are understudied.

**Objective:**

To investigate the metabolic associations within the maternal-placental-fetal unit and subsequent neurodevelopmental outcomes in younger siblings of children with autism spectrum disorder (ASD).

**Design, Setting, and Participants:**

This cohort study was conducted within a subset of the Markers of Autism Risk in Babies, Learning Early Signs (MARBLES) cohort. MARBLES is a prospective birth cohort of younger siblings of children with ASD assessed for neurodevelopmental outcomes at approximately age 36 months. Participants in MARBLES were recruited through the UC Davis MIND Institute. This subset of the MARBLES cohort included younger siblings born between 2009 and 2015. Maternal third trimester serum, placental tissue, and umbilical cord serum samples were collected from participants. Only pregnancies with at least 2 of these sample types were included in this analysis. Data analysis was conducted from March 1, 2023, to March 15, 2024.

**Exposures:**

Quantitative metabolomics analysis was conducted on maternal third trimester serum, as well as placental tissue and umbilical cord serum collected at delivery.

**Main Outcomes and Measures:**

Using the Autism Diagnostic Observation Schedule and Mullen Scales of Early Learning, outcomes were classified as ASD, other nontypical development (non-TD), and typical development (TD).

**Results:**

This analysis included 100 maternal serum samples, 141 placental samples, and 124 umbilical cord serum samples from 152 pregnancies (median [IQR] maternal age, 34.6 [30.8-38.3] years; median [IQR] gestational age, 39.0 [38.6-39.7] weeks; 87 [57.2%] male infants). There was no evidence that the maternal third trimester serum metabolome was significantly associated with the other metabolomes. The placental and cord serum metabolomes were highly correlated (first latent variate pair: *R^2^* = 0.75; *P* < .001) and the variate scores for each tissue were significantly associated with reduced risk of non-TD (placenta: relative risk [RR], 0.13; 95% CI, 0.02-0.71; cord: RR, 0.13; 95% CI, 0.03-0.70) but not ASD (placenta: RR, 1.09; 95% CI, 0.42-2.81; cord: RR, 0.63; 95% CI, 0.23-1.73) compared with the TD reference group.

**Conclusions and Relevance:**

In this cohort study of children with high familial risk of ASD, placental and cord serum metabolism at delivery were highly correlated. Furthermore, placental and cord serum metabolic profiles were associated with risk of non-TD.

## Key Points

**Question:**

Are metabolomes in the maternal-placental-fetal unit associated with each other and subsequent neurodevelopmental outcomes?

**Findings:**

In this cohort study of 100 maternal serum samples, 141 placental samples, and 124 umbilical cord serum samples from 152 pregnancies of younger siblings of children with autism spectrum disorder (ASD), multivariate analysis revealed that the placental and cord serum metabolomes were significantly correlated. Placental and cord serum latent variates were significantly associated with reduced risk of nontypical development but not ASD.

**Meaning:**

These findings in a cohort with high familial ASD risk, placental and umbilical cord metabolism at birth was associated with neurodevelopmental outcomes.

## Teaser

This cohort study examines maternal, placental, and fetal metabolites in association with each other and with risk of autism spectrum disorder or nontypical development among younger siblings of children with autism spectrum disorder.

## Introduction

During pregnancy, many maternal physiological and metabolic adaptions occur to ensure healthy development,^1^ including maternal insulin resistance and elevated circulating lipids to support fetal growth.^2^ Early disturbances in maternal metabolism during pregnancy are associated with child outcomes, including neurodevelopment.^3,4,5^ In a targeted analysis of maternal plasma methylation cycle and transsulfuration pathway metabolites, prenatal metabolic profiles differed significantly between participants with low and high familial risk of having a subsequent child with autism spectrum disorder (ASD).^4^ In a case-control study, untargeted midpregnancy serum metabolomics identified pathways associated with ASD, including metabolism of glycosphingolipids, phosphatidylinositol phosphate, and steroid hormones.^5^ These disturbances may reflect altered nutrient availability during sensitive developmental periods that support rapid brain growth during the third trimester.^6^

Just as changes in maternal circulating metabolites are associated with fetal development, so too are changes in placental metabolism. All fetal nutrition must pass through the placenta, and alterations in placental function are likewise associated with fetal neurodevelopmental outcomes.^7,8^ Umbilical cord blood has also been used to investigate fetal metabolism at birth.^9^ However, we are not aware of any analysis of the associations among maternal, placental, and fetal metabolism. Therefore, we aimed to investigate the associations among metabolism in maternal serum, placental tissue, and umbilical cord serum using quantitative metabolomics and whether these measures were associated with neurodevelopmental outcomes in a prospective pregnancy cohort with high familial risk of ASD.

## Methods

The University of California, Davis, institutional review board and the California Committee for the Protection of Human Subjects approved this study and the Markers of Autism Risk in Babies, Learning Early Signs (MARBLES) study protocols. All participants provided written informed consent for collection of data and specimens. We followed the Strengthening the Reporting of Observational Studies in Epidemiology (STROBE) reporting guideline for cohort studies. Data analysis was conducted from March 1, 2023, to March 15, 2024.

### Study Participants, Sample Preparation, and Quantitative Nuclear Magnetic Resonance Metabolomics Analysis

Samples analyzed in this study were collected through the MARBLES study, a prospective birth cohort following younger siblings of children with ASD.^10^ We selected samples from MARBLES pregnancies with at least 2 of the following samples available: maternal third trimester serum, placenta collected at delivery, or umbilical cord serum collected at delivery. Some mothers were enrolled in MARBLES through multiple pregnancies, so we limited our analysis to the first available pregnancy that met these criteria to ensure the pregnancies studied were independent within each analysis. A total of 100 maternal third-trimester serum samples, 141 placental samples, and 124 cord serum samples were included in this analysis (eFigure 1 in Supplement 1). There were 89 mother-infant dyads for whom a maternal serum sample and placental sample were available, 72 mother-infant dyads for whom a maternal serum sample and a cord blood sample were available, and 113 mother infant–dyads for whom a placental sample and a cord serum sample were available. However, only 111 mother-infant dyads were included in the placenta-cord serum analysis, since 2 infants were excluded for having an older sibling included in the analysis. Of these, there were 61 mother-infant dyads with all sample types available.

At approximately age 36 months, neurodevelopment was evaluated by MARBLES clinicians using the Mullen’s Scales of Early Learning and ASD Diagnostic Observation Schedule (ADOS).^11,12^ Neurodevelopment was classified as described elsewhere^13^: ASD was diagnosed when ADOS scores met or exceeded the ASD cutoff; nontypical development (non-TD) was diagnosed when ADOS scores were within 3 points below the ASD cutoff, or when Mullen’s Scales of Early Learning scores and subdomain scores were 1.5 to 2 SDs below the mean; and typical development (TD) was diagnosed when criteria for ASD or non-TD were not met.

Whole blood was collected during the third trimester and umbilical cord blood was collected at delivery. After collection, blood was centrifuged, and the resulting serum was collected and stored at −80 °C until preparation for metabolomics analysis. Samples were prepared for metabolomics analysis by thawing on ice and subsequently subjected to ultrafiltration centrifugation to remove protein using Amicon Ultra-0.5 mL 3000 MW centrifugal filters (Millipore), as described elsewhere.^14^ For maternal serum, filtrate volume was adjusted to 207 μL with ultrapure water if an insufficient sample was collected, and an internal standard, containing 5.0 mmol/L 3-(trimethylsilyl)-1-propanesulfonic acid-d6 (DSS-d6), 0.2% sodium azide, and 99.8% deuterium oxide (Chenomx) was added. For umbilical cord serum, filtrate volumes were measured, and filtrates were frozen, dried using a miVac concentrator system (Genevac), and reconstituted using 240 μL of 10 mmol/L potassium phosphate buffer prepared in deuterium oxide to improve signal-to-noise. The pH of each sample was adjusted to 6.8 (±0.1) and 180 μL was loaded into 3-mm nuclear magnetic resonance tubes (Bruker) and stored at 4 °C until spectral acquisition.

Placentas were processed at delivery, with full-thickness sections of tissue collected and stored at −80 °C. For metabolomics analysis, samples were partially thawed and 6-mm biopsy punches were collected for metabolic analyses, as described elsewhere.^14^ Placenta tissue was cryoground using liquid nitrogen and approximately 80 mg of tissue was weighed and extracted using a 2-step chloroform methanol water extraction.^15^ The upper layer was collected, measured, frozen, dried using a miVac concentrator system, and subsequently stored at −80 °C until preparation for proton nuclear magnetic resonance spectroscopy. Dried samples were reconstituted in 10 mmol/L potassium phosphate buffer, the pH was adjusted to 6.8 (±0.1), and an internal standard was added as described.

An Avance 600-MHz spectrometer (Bruker) equipped with a SampleJet was used to acquire proton nuclear magnetic resonance spectra as previously described.^16^ Spectra were manually phase- and baseline-corrected, and metabolite concentrations were quantified using Chenomx software version 8.1 (Chenomx). This process relies on a library of spectral signatures for small molecules and the internal standard, DSS-d6. The library allows for identification of metabolites through matching the spectral signature, while the internal standard allows for determination of the concentration of each metabolite within the spectrum.^17^ This method has been shown to be both accurate and reproducible.^18,19^ Metabolites that might have been introduced during sample preparation or that were identified in less than 80% of samples were excluded from statistical analysis, as described elsewhere.^14^

### Covariate Selection

We included a minimal model (model 1) and a fully adjusted model (model 2) for each analysis (eTable 1 in Supplement 1). Model 1 covariates were selected a priori as factors related to sample collection and storage, including birth year, gestational age at sample collection, and fasted time at sample collection (for maternal serum samples). We used a directed acyclic graph to identify sufficient covariate adjustment sets for our analysis of the associations among the maternal serum, placental, and cord serum metabolomes (eFigure 2 in Supplement 1) using the R package dagitty.^20^ Variables used as proxies for social and economic inequities, such as maternal race and ethnicity, education, and home ownership, were collected by self-report shortly after enrollment using a standardized questionnaire. Race and ethnicity were categorized as White or historically marginalized group, including Asian; Black or African American; Hispanic, non-White; Hispanic, White; Pacific Islander; and multiracial. We also used directed acyclic graphs to identify covariate adjustment sets to evaluate the associations between metabolism and neurodevelopment (eFigure 3 in Supplement 1). In these models, we collapsed maternal metabolic condition into a dichotomous variable, defined as prepregnancy body mass index (BMI; calculated as weight in kilograms divided by height in meters squared) less than 25 and no metabolic conditions (reference group) or prepregnancy BMI 25 or greater or any diabetes or hypertensive disorder, to reduce the number of small cells.

### Statistical Analysis

Correlations between metabolites within each tissue (maternal serum, placenta, and cord serum) were evaluated with Spearman correlations, and *P* values were corrected for false discovery rate (FDR) using the Benjamini-Hochberg procedure (eFigures 4-6 in Supplement 1).^21^ Bipartite Spearman correlations were used to evaluate the association between each pair of metabolomes and *P* values were FDR corrected (eFigures 7-9 in Supplement 1). To adjust for covariates, we used rank-based linear regression. We report FDR *q* values and considered 2-sided *q* < .10 significant.

When metabolites in 1 metabolome (matrix X) were significantly associated with metabolites in a second metabolome (matrix Y), we used partial least squares (PLS) for multivariate analysis and dimension reduction after partialling out the associations from covariates in model 2. When the number of mother-infant dyads was less than the sum of the metabolites in the 2 metabolomes, sparse PLS was used and tuned using the R package *mixOmics*.^22^ Since maternal serum, placenta, and cord serum can affect each other, we used canonical PLS, which seeks to model bidirectional associations between X and Y.^23^ The number of latent variate pairs retained in the model was selected using the coefficient of prediction (*Q^2^*) in leave-1-out cross-validation. We then determined if the covariance between the latent variate pairs was greater than by chance alone using permutation testing with 9999 permutations. When the models were better than chance, the results were visualized using relevance networks showing bipartite associations between the 2 metabolomes.^23^ We used multinomial logistic regression to evaluate the association between metabolism and neurodevelopmental outcomes.

All statistical analyses were conducted in RStudio version 2022.12.0 using the R statistical language version 4.3.1 (R Project for Statistical Computing). Rank-based linear regressions were implemented using *Rfit*.^24^ Multinomial logistic regression models were implemented using *nnet* and estimated probabilities were simulated using *MNLpred*.^25,26^ All visualizations were made using *ggplot2*, *ggdag*, and *igraph*.^27,28,29^

## Results

### Study Population

This analysis included 152 total pregnancies (median [IQR] maternal age, 34.6 [30.8-38.3] years; median [IQR] gestational age, 39.0 [38.6-39.7] weeks), including 87 (57.2%) male and 65 (42.8%) female children. Forty-five children met the diagnostic criteria for ASD, 19 children were classified as non-TD, 87 children were classified as TD, and 1 child was missing a neurodevelopmental outcome. Descriptive statistics by neurodevelopmental outcome for the mother-infant dyads in each analysis are presented in Table 1.

**Table 1.** Demographic Characteristics of Mother-Infant Dyads

Characteristic	Maternal								Placenta-cord serum (n = 111)			
	Serum-placenta (n = 89)				Serum-cord serum (n = 71)^a^							
	Participants, No. (%)			*P* value^b^	Participants, No. (%)			*P* value^b^	Participants, No. (%)			*P* value^b^
	TD (n = 54)	ASD (n = 25)	Non-TD (n = 10)		TD (n = 40)	ASD (n = 23)	Non-TD (n = 8)		TD (n = 61)	ASD (n = 37)	Non-TD (n = 13)	
Birth year, median (IQR)	2011 (2009-2012)	2011 (2010-2012)	2011 (2009-2013)	.98	2011 (2010-2013)	2011 (2010-2012)	2012 (2010-2013)	.98	2011 (2010-2014)	2012 (2010-2014)	2013 (2010-2014)	.61
Gestational age at serum collection, median (IQR), d	236 (221-249)	230 (215-242)	246 (220-250)	.47	237 (224-251)	239 (220-246)	248 (223-251)	.72	NA	NA	NA	NA
Time since last meal or snack at serum collection, median (IQR), min	74 (30-124)	85 (55-135)	84 (52-104)	.48	74 (39-124)	105 (52-140)	85 (49-113)	.77	NA	NA	NA	NA
Gestational age at delivery, median (IQR),wk	39 (38-40)	39 (39-40)	39 (39-39)	.26	39 (38-40)	39 (39-40)	39 (38-39)	.31	39 (38-40)	39 (39-40)	39 (39-39)	.03
Birth weight, median (IQR), g	3405 (3170-3675)	3395 (3290-3595)	3272 (3151-3322)	.30	3372 (3184-3638)	3395 (3315-3650)	3308 (3230-3370)	.43	3493 (3195-3755)	3374 (3290-3706)	3311 (3240-3500)	.59
Delivery payer												
Private insurance	42 (78)	19 (76)	6 (67)	.77	31 (76)	16 (70)	4 (57)	.49	48 (79)	24 (65)	6 (50)	.08
Government program	12 (22)	6 (24)	3 (33)		9 (23)	7 (30)	3 (43)		13 (21)	13 (35)	6 (50)	
Missing	0	0	1		0	0	1		0	0	1	
Delivery method												
Vaginal	29 (57)	17 (68)	7 (70)	.54	23 (61)	15 (65)	7 (88)	.35	32 (54)	28 (76)	7 (54)	.09
Cesarean	22 (43)	8 (32)	3 (30)		15 (39)	8 (35)	1 (13)		27 (46)	9 (24)	6 (46)	
Missing	3	0	0		2	0	0		2	0	0	
Mother’s age at delivery, median (IQR), y	36 (33-39)	36 (32-40)	35 (34-37)	.84	35 (31-39)	35 (30-39)	34 (30-35)	.41	34 (32-39)	34 (30-38)	34 (29-37)	.68
Mother’s race and ethnicity												
Historically marginalized group				.70				.95				.95
Any	25 (46)	13 (52)	6 (60)		18 (45)	10 (43)	4 (50)		30 (49)	18 (49)	7 (534)	
Asian	10 (19)	5 (20)	2 (20)		6 (15)	4 (17)	1 (13)		8 (13)	4 (11)	2 (15)	
Black/African-American	1 (2)	1 (4)	1 (10)		0	1 (4)	1 (13)		0	3 (8)	1 (8)	
Hispanic, non-White	0	1 (4)	1 (10)		2 (5)	1 (4)	0		1 (2)	1 (2.7)	0	
Hispanic, White	12 (22)	5 (20)	2 (20)		10 (25)	4 (17)	2 (25)		17 (28)	7 (19)	4 (31)	
Pacific Islander	0	0	0		0	0	0		1 (2)	0	0	
Multiracial	2 (4)	1 (4)	0		0	0	0		3 (5)	3 (8)	0	
White	29 (54)	12 (48)	4 (40)		22 (55)	13 (57)	4 (50)		31 (51)	19 (51)	6 (46)	
Mother’s education^c^												
≤Some college	26 (48)	12 (48)	6 (60)	.78	17 (42)	13 (57)	5 (63)	.41	26 (43)	25 (68)	7 (54)	.06
<High school or GED	2 (4)	1 (4)	0		1 (3)	1 (4.3)	0		2 (3)	2 (5)	0	
High school or GED	4 (7)	1 (4)	1 (10)		2 (5)	1 (4)	0		3 (5)	3 (8)	0	
Some college	20 (37)	10 (40)	5 (50)		14 (35)	11 (48)	5 (63)		21 (34)	20 (54)	7 (54)	
≥Bachelor’s degree	28 (52)	13 (52)	4 (40)		23 ()	10 (43)	3 (38)		35 (57)	12 (32)	6 (46)	
Bachelor’s degree	21 (39)	8 (32)	2 (20)		17 (42)	6 (26)	2 (25)		22 (36)	6 (16)	2 (15)	
Graduate or professional degree	7 (13)	5 (20)	2 (20)		6 (15)	4 (17)	1 (13)		13 (21)	6 (16)	4 (31)	
Maternal metabolic condition												
BMI <25, no metabolic conditions	20 (38)	7 (28)	5 (50)	.40	13 (33)	7 (30)	4 (50)	.58	24 (41)	10 (27)	5 (38)	.17
BMI <30, no metabolic conditions	10 (19)	6 (24)	3 (30)		10 (26)	6 (26)	2 (25)		12 (20)	13 (35)	3 (23)	
BMI >30, no metabolic conditions	6 (11)	4 (16)	0		7 (18)	4 (17)	0		12 (20)	6 (16)	1 (8)	
Any hypertensive disorder without diabetes, at any BMI	1 (2)	3 (12)	1 (10)		0	2 (9)	1 (13)		0	3 (8)	2 (15)	
Any diabetes, at any BMI	16 (30)	5 (20)	1 (10)		9 (23)	4 (17)	1 (13)		11 (19)	5 (14)	2 (15)	
Missing	1	0	0		1	0	0		2	0	0	
Parental homeownership												
No	25 (46)	9 (38)	4 (44)	.77	17 (43)	8 (38)	5 (63)	.49	27 (44)	18 (50)	9 (69)	.26
Yes	29 (54)	15 (63)	5 (56)		23 (58)	13 (62)	3 (38)		34 (56)	18 (50)	4 (31)	
Missing	0	1	1		0	2	0		0	1	0	
Prenatal vitamin use in the first month of pregnancy												
No	29 (54)	15 (60)	5 (56)	.87	15 (38)	14 (64)	4 (50)	.14	25 (42)	25 (68)	8 (62)	.04
Yes	25 (46)	10 (40)	4 (44)		25 (63)	8 (36)	4 (50)		35 (58)	12 (32)	5 (38)	
Missing	0	0	1		0	1	0		1	0	0	
Child’s sex												
Male	30 (56)	16 (64)	3 (30)	.19	24 (60)	15 (65)	4 (50)	.75	35 (57)	26 (70)	4 (31)	.04
Female	24 (44)	9 (36)	7 (70)		16 (40)	8 (35)	4 (50)		26 (43)	11 (30)	9 (69)	

Abbreviations: BMI, body mass index (calculated as weight in kilograms divided by height in meters squared); GED, general educational development; NA, not applicable.

^a^ Note that 1 child in the maternal serum-cord serum analysis had a missing neurodevelopmental diagnosis.

^b^ Differences by neurodevelopmental outcomes were tested using the Kruskal-Wallis test for continuous variables and χ^2^ test for categorical variables.

^c^ Maternal education was originally collected as a variable with 5 levels. For this analysis, education was collapsed into a 2-level variable as some college or less and Bachelor’s degree or higher. The original variables levels are presented here for completeness.

### Maternal Serum and Placental Metabolomes

Bipartite Spearman correlations revealed no significant associations among 48 maternal third trimester serum metabolites and 54 placental metabolites after FDR correction (eFigure 7 in Supplement 1). Likewise, rank-based regression adjusted for covariates did not identify a significant association between any metabolite pair after FDR correction (eTable 2 in Supplement 2). PLS was not conducted because no significant associations were found between maternal serum metabolites and placental metabolites.

### Maternal Serum and Umbilical Cord Serum Metabolomes

Bipartite Spearman correlations corrected for FDR revealed few significant associations among 48 maternal third trimester serum metabolites and 44 umbilical cord serum metabolites (eFigure 8 in Supplement 1). Rank-based regression adjusted for covariates in models 1 and 2 identified similar patterns after FDR correction (Table 2). The associations between other metabolite pairs with *q* > .10 are presented in eTable 3 in Supplement 2.

**Table 2.** Estimates of the Associations of Each Maternal Third Trimester Serum Metabolite With Each Umbilical Cord Serum Metabolite

Maternal serum metabolite	Cord serum metabolite	Model 1^a^			Model 2^b^		
		Estimate (SE)	*P* value	FDR *q* value^c^	Estimate (SE)	*P* value	FDR *q* value^c^
Betaine	Creatine	−0.51 (0.14)	.001	.15	−0.57 (0.15)	<.001	.08
Creatinine	Creatinine	0.53 (0.09)	<.001	<.001	0.54 (0.096)	<.001	<.001
Dimethyl sulfone	Dimethyl sulfone	0.92 (0.10)	<.001	<.001	1.00 (0.10)	<.001	<.001
Glycine	Glycine	0.28 (0.06)	<.001	.02	0.27 (0.06)	<.001	.07
Glycine	Isoleucine	−0.31 (0.08)	<.001	.08	−0.26 (0.07)	<.001	.08
Threonine	Threonine	0.33 (0.05)	<.001	<.001	0.33 (0.06)	<.001	<.001

Abbreviation: FDR, false discovery rate.

^a^ Model 1 was adjusted for birth year, gestational age at maternal serum collection, fasted time at maternal serum collection, and gestational age at umbilical cord serum collection (delivery).

^b^ Model 2 was adjusted for all covariates in model 1 and maternal education, maternal race and ethnicity, home ownership, and maternal metabolic conditions.

^c^ All *P* values were adjusted for FDR and both the original *P* values and FDR quantities, *q* values, are reported. Only findings with FDR *q* < .10 are reported here.

With few significant associations between individual metabolites, we did not expect to find a significant multivariate association between the maternal and cord serum metabolomes. When we used sparse PLS to test this multivariate association (adjusted for model 2 covariates), the model failed to converge under a variety of tuning conditions, indicating poor correlation between the 2 metabolomes. The covariance between the first latent variate pair was not significantly different from chance (covariance, 0.04; *P* = .27).

### Placental and Umbilical Cord Serum Metabolomes

Bipartite Spearman correlations revealed many associations between 54 placental metabolites and 44 cord serum metabolites after FDR correction, particularly among lipid-, energy-, and amino acid–related metabolites (eFigure 9 in Supplement 1). Rank-based regression adjusted for covariates identified similar patterns after FDR correction (Table 3). The findings between other metabolite pairs with *q* > .10 are presented in eTable 4 in Supplement 2.

**Table 3.** Estimates of the Associations Between Each Placental Metabolite and Each Umbilical Cord Serum Metabolite

Placenta metabolite	Cord Serum metabolite	Model 1^a^			Model 2^b^		
		Estimate (SE)	*P* value	FDR *q* value^c^	Estimate (SE)	*P* value	FDR *q* value^c^
2-Hydroxybutyrate	2-Aminobutyrate	0.40 (0.06)	<.001	<.001	0.37 (0.06)	<.001	<.001
2-Hydroxybutyrate	2-Hydroxybutyrate	0.81 (0.04)	<.001	<.001	0.80 (0.04)	<.001	<.001
2-Hydroxybutyrate	2-Oxoisocaproate	0.27 (0.05)	<.001	<.001	0.26 (0.04)	<.001	<.001
2-Hydroxybutyrate	3-Hydroxybutyrate	1.04 (0.21)	<.001	.001	1.09 (0.22)	<.001	.001
2-Hydroxybutyrate	3-Hydroxyisobutyrate	0.23 (0.04)	<.001	<.001	0.20 (0.04)	<.001	.001
2-Hydroxybutyrate	3-Methyl-2-oxovalerate	0.17 (0.05)	.001	.08	0.11 (0.04)	.014	.34
2-Hydroxybutyrate	Proline	−0.14 (0.04)	.002	.13	−0.15 (0.04)	.001	.09
3-Hydroxybutyrate	2-Hydroxybutyrate	0.22 (0.04)	<.001	<.001	0.21 (0.04)	<.001	<.001
3-Hydroxybutyrate	3-Hydroxybutyrate	0.99 (0.03)	<.001	<.001	1.01 (0.03)	<.001	<.001
3-Hydroxybutyrate	3-Methyl-2-oxovalerate	0.08 (0.02)	<.001	.05	0.07 (0.02)	<.001	.04
3-Hydroxybutyrate	Alanine	−0.07 (0.02)	.001	.07	−0.07 (0.02)	.001	.08
3-Hydroxybutyrate	Asparagine	−0.06 (0.02)	.001	.07	−0.07 (0.02)	<.001	.03
Arginine	Lysine	0.16 (0.04)	<.001	.05	0.17 (0.04)	<.001	.02
Asparagine	Histidine	0.14 (0.04)	.001	.08	0.17 (0.04)	<.001	.01
Asparagine	Threonine	0.22 (0.06)	<.001	.04	0.23 (0.06)	<.001	.02
Aspartate	2-Aminobutyrate	0.35 (0.10)	.001	.07	0.25 (0.11)	.03	.42
Betaine	Betaine	0.23 (0.05)	<.001	.001	0.23 (0.04)	<.001	<.001
Isoleucine	Histidine	0.13 (0.05)	.005	.21	0.16 (0.05)	.001	.09
Lactate	Histidine	0.16 (0.06)	.006	.22	0.21 (0.06)	.001	.06
Lactate	Ornithine	0.43 (0.11)	.0002	.04	0.43 (0.13)	.001	.097
Lysine	Lysine	0.20 (0.04)	<.001	.001	0.19 (0.05)	<.001	.01
Lysine	Threonine	0.24 (0.06)	<.001	.02	0.22 (0.06)	<.001	.04
Methionine	Histidine	0.14 (0.04)	.002	.13	0.17 (0.05)	<.001	.03
Methionine	Threonine	0.23 (0.06)	<.001	.05	0.24 (0.06)	<.001	.03
Pantothenate	Isoleucine	−0.11 (0.03)	.001	.07	−0.09 (0.03)	.01	.30
Threonine	Threonine	0.41 (0.05)	<.001	<.001	0.40 (0.06)	<.001	<.001
Tyrosine	Histidine	0.13 (0.05)	.006	.22	0.16 (0.05)	.001	.08
Tyrosine	Threonine	0.21 (0.06)	.001	.11	0.21 (0.06)	.001	.08
Tyrosine	Tyrosine	0.19 (0.06)	.001	.10	0.22 (0.06)	<.001	.03
Uracil	Methionine	0.11 (0.03)	.002	.13	0.12 (0.03)	<.001	.05
myo-Inositol	Histidine	0.12 (0.04)	.005	.21	0.17 (0.04)	<.001	.01

Abbreviation: FDR, false discovery rate.

^a^ Model 1 was adjusted for birth year and gestational age at sample collection (delivery).

^b^ Model 2 was adjusted for all covariates in model 1 and fetal sex, maternal education, maternal race and ethnicity, home ownership, prenatal vitamin use in the first month of pregnancy, and maternal metabolic conditions.

^c^ All *P* values were adjusted for FDR and both the original *P* values and FDR quantities, *q* values, are reported. Only findings with FDR *q* < .10 are reported here.

With many significant associations observed between individual metabolites, we used PLS to test the association among the concentrations of 54 placental metabolites and 44 cord serum metabolites from 107 mother-infant dyads with complete model 2 covariates, retaining 1 latent variate pair after leave-1-out cross-validation. The first latent variate pair explained 74.7% of the variance, and covariance between the first latent variate pair was significantly different from chance (covariance, 0.15; *P* < .001). The relevance network revealed central roles for placental and cord serum 3-hydroxybutyrate (3-OHB) related to lipid, energy, and amino acid metabolism (Figure, A). Furthermore, both placental and cord serum 3-OHB concentrations were most strongly associated with the placental and cord serum latent variates, respectively (eFigure 10 in Supplement 1).

**Figure.**
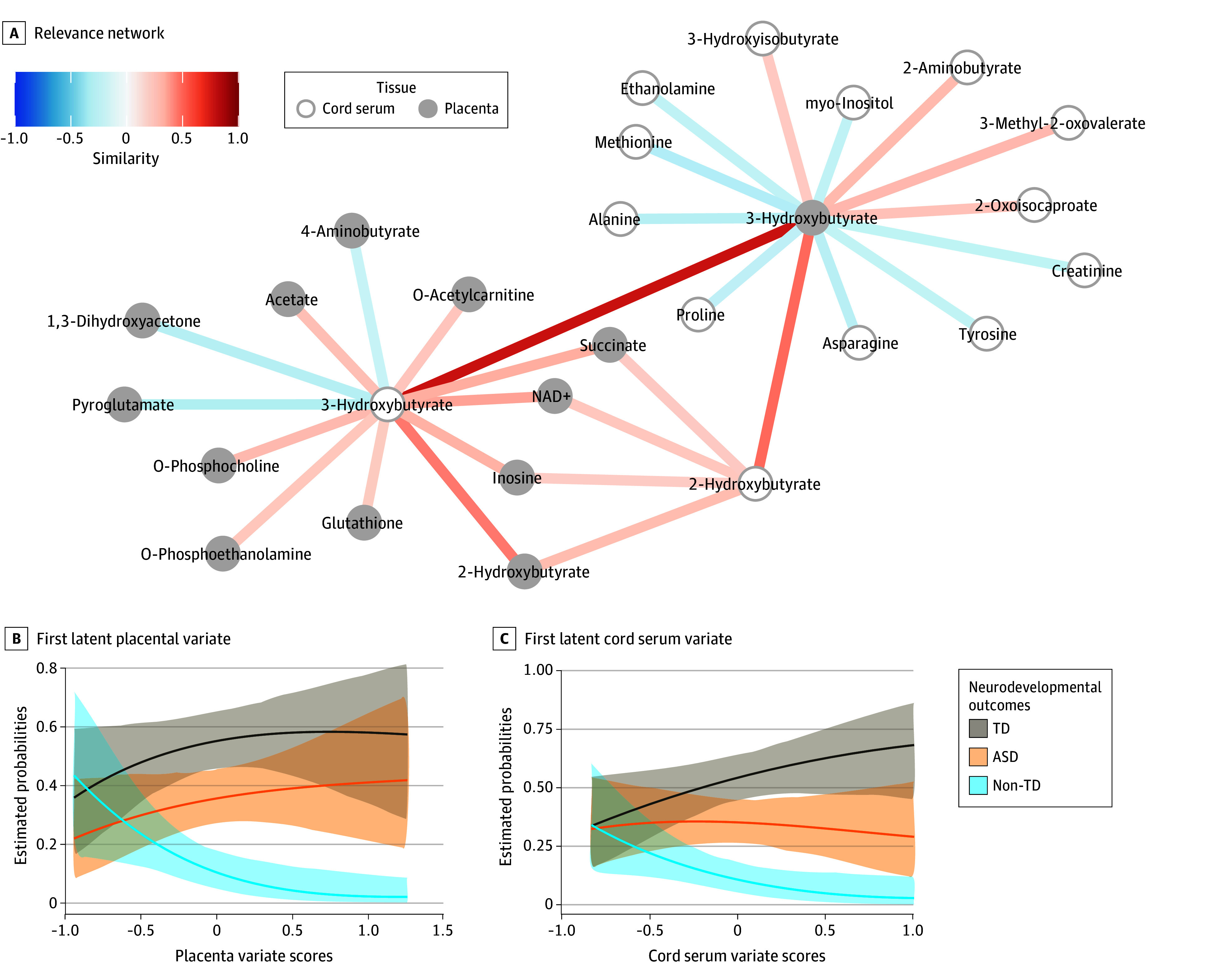
Placenta-Cord Serum Metabolic Profiles and Neurodevelopment A, The relevance network shows bipartite similarity scores between cord serum metabolites and placental metabolites. Metabolites are represented by nodes: gray for placental metabolites and white for cord serum metabolites. Edges can only connect nodes from different tissues. Positively associated metabolites are joined by red edges, while negatively associated metabolites are joined by blue edges. Associations between the variate scores and neurodevelopment were visualized as estimated probabilities and 95% CIs for neurodevelopmental outcomes with increasing scores for (B) the first latent placental variate and (C) the first latent cord serum variate.

Since the model 2 covariates were also appropriate to evaluate the association between the first latent variate pair and neurodevelopment (eFigure 3 in Supplement 1), multinomial logistic regression was used to evaluate ASD and non-TD risk compared with TD references (Figure, B and C). First placenta latent variate scores were associated with reduced risk of non-TD (relative risk [RR], 0.13; 95% CI, 0.02-0.71; *P* = .02) but not ASD (RR, 1.09; 95% CI, 0.45-3.01; *P* = .76). First cord serum latent variate scores were associated with reduced risk of non-TD (RR, 0.13; 95% CI, 0.03-0.70; *P* = .02) but not ASD (RR, 0.63; 95% CI, 0.23-1.73; *P* = .37).

### 3-OHB and Neurodevelopment

Since 3-OHB was the largest driver of the placental and cord variate scores, we tested associations of 3-OHB concentrations with neurodevelopmental outcomes (Table 4). In unadjusted and adjusted models, cord 3-OBH was positively associated with risk of non-TD (RR, 9.03; 95% CI, 1.52-53.71; *P* = .02; adjusted RR [aRR], 8.88; 95% CI, 1.23-64.05; *P* = .03), but not ASD (RR, 0.91; 95% CI, 0.32-2.61; *P* = .86; aRR, 1.45; 95% CI, 0.42-4.98; *P* = .56). There was no significant association of placental 3-OHB with risk of non-TD (RR, 5.21; 95% CI, 0.92-29.44; *P* = .06; aRR, 5.26; 95% CI, 0.76-36.24; *P* = .09) or ASD (RR, 0.64; 95% CI, 0.23-1.84; *P* = .41; aRR, 1.02; 95% CI, 0.30-3.24; *P* = .97). In the unadjusted and adjusted models, serum 3-OHB was associated with reduced risk of non-TD (RR, 0.06; 95% CI, 0.00-0.76; *P* = .03; aRR, 0.03; 95% CI, 0.00-0.77; *P* = .03) but was not associated with risk of ASD (RR, 0.24; 95% CI, 0.05-1.25; *P* = .09; aRR, 0.27; 95% CI, 0.04-1.71; *P* = .16).

**Table 4.** Unadjusted and Adjusted Associations of Maternal Serum, Placental, and Umbilical Cord Serum 3-Hydroxybutyrate With Neurodevelopmental Outcome

Tissue	3-Hydroxybutyrate, median (range)			Unadjusted				Adjusted			
				ASD vs TD		Non-TD vs TD		ASD vs TD		Non-TD vs TD	
	TD	ASD	Non-TD	RR (95% CI)	*P* value	RR (95% CI)	*P* value	RR (95% CI)	*P* value	RR (95% CI)	*P* value
Maternal serum, nmol/L	50.2 (11.2-268.9)	38.6 (12.9-174.5)	30 (16.9-99.1)	0.24 (0.05-1.25)	.09	0.06 (0.00-0.76)	.03	0.27 (0.04-1.71)^a^	.16	0.03 (0.00-0.77)^a^	.03
Cord serum, nmol/L	139.8 (14.7-997)	121.6 (21-470.5)	259.3 (64.7-651.5)	0.91 (0.32-2.61)	.86	9.03 (1.52-53.71)	.02	1.45 (0.42-4.98)^b^	.56	8.88 (1.23-64.05)^b^	.03
Placenta, nmol/g	150.5 (17.8-1154.8)	141.5 (13.6-541.9)	253.2 (55.0-778.8)	0.64 (0.23-1.84)	.41	5.21 (0.92-29.44)	.06	1.02 (0.30-3.42)^c^	.97	5.26 (0.76-36.24)^c^	.09

Abbreviations: ASD, autism spectrum disorder; non-TD, non-typical development; RR, relative risk; TD, typical development.

^a^ Adjusted for gestational age at sampling, fasted time at sampling, fetal sex, home ownership, maternal education, maternal race and ethnicity, and maternal metabolic condition as a dichotomous variable.

^b^ Adjusted for year of birth, gestational age at birth, delivery mode, prenatal vitamin use in the first month of pregnancy, fetal sex, home ownership, maternal race and ethnicity, and maternal education and maternal metabolic condition as a dichotomous variable.

^c^ Adjusted for year of birth, gestational age at birth, delivery mod, prenatal vitamin use in the first month of pregnancy, fetal sex, home ownership, maternal race and ethnicity, and maternal education and maternal metabolic condition as a dichotomous variable.

## Discussion

In this cohort study investigating the associations among maternal, placental, and fetal metabolism, we found that the placental and umbilical cord serum metabolomes were highly correlated. We also found that 3-OHB was an important metabolite across the maternal, placental, and fetal metabolomes and that lower levels of 3-OHB in maternal serum and higher levels in cord serum were associated with increased risk of non-TD. We speculate that these unexpected findings are related to a metabolic switch that occurs during the perinatal transition.^30^ Labor is a highly coordinated process, associated with changes in maternal and fetal metabolism.^31,32^ Birth itself is associated with profound metabolic and transcriptomic changes in the newborn to maintain energy supply after losing the steady supply of maternal nutrients.^33,34^ The placental and cord serum metabolomes were collected at the same postdelivery time point and likely reflect profound metabolic changes in this critical window, whereas maternal serum collected during the third trimester reflects a different developmental window. It is possible that metabolic profiles in maternal serum collected perinatally would more closely align with placental-fetal metabolic profiles. Additionally, the opposite association between non-TD risk and maternal 3-OHB could be explained by these different developmental windows.

In maternal serum, the negative association between 3-OHB and non-TD risk could relate to lipid metabolism. During the third trimester, maternal insulin resistance and lipolysis ensures nutrient availability for the placenta and developing fetus.^35^ High free fatty acids in the blood trigger the production of ketones, which readily cross the placenta to be used as fuel or substrate for lipid and cholesterol synthesis.^36^ Indeed, maternal 3-OHB has been shown to be rapidly incorporated into placental cholesterol and fetal liver and brain tissues in a rat model.^37^ Lower maternal 3-OHB concentrations in the third trimester might indicate dysregulated maternal lipid metabolism, resulting in reduced availability of important lipid substrates for brain development. While this analysis was limited to the polar metabolome, a 2021 study^38^ reporting on the untargeted metabolomic analysis of maternal third trimester plasma showed that higher levels of fatty acids involved in lipid biosynthesis were associated with reduced risk of non-TD in the MARBLES cohort. Taken together, these findings suggest that disturbed lipid metabolism during late pregnancy could play a role in the etiology of non-TD.

Lipid metabolism is disrupted in gestational diabetes and preeclampsia,^35^ and both of these conditions are risk factors associated with non-TD.^39^ Interestingly, negative associations between third trimester maternal circulating 3-OHB concentrations and Bayley Scales of Infant and Toddler Development mental development index scores at age 2 years and mean Stanford-Binet Intelligence Scale scores during preschool age have been reported by Rizzo et al.^40^ However, the study by Rizzo et al^40^ reported much higher levels of plasma 3-OHB for healthy women than reported here. This could reflect differences in fasting status between these studies: most MARBLES participants had eaten in the 2 hours before blood collection, while participants in the study by Rizzo et al^40^ fasted overnight. However, these findings might also indicate a U-shaped association between altered neurodevelopment with maternal third trimester circulating 3-OHB.

The placental and cord serum metabolomes could reflect the metabolic adaptations to labor and birth.^33^ Here, we observed and association between cord serum 3-OHB concentrations with metabolites related to lipid, tricarboxylic acid cycle, and amino acid catabolism pathways. This aligns with metabolic changes at birth, when the steady supply of maternal glucose is abruptly cut off and the newborn must rely on gluconeogenesis and ketogenesis until feeding is initiated.^33^ The newborn also shifts to oxidative metabolism in the new oxygen-rich extrauterine environment.^41^ Increased oxidative metabolism can lead to increased oxidative stress. Indeed, we observed that cord serum and placental 3-OHB were positively associated with cord serum and placental 2-hydroxybutyrate (2-OHB) and 2-aminobutyrate, biomarkers of glutathione synthesis and status.^42,43^ Cord serum 3-OHB was also negatively associated with pyroglutamate, a metabolite related to glutathione depletion^44,45^ and positively associated with placental glutathione concentrations. 3-OHB acts as a signaling molecule to protect against oxidative stress through several proposed mechanisms, including activation of antioxidant response-related transcription factors.^46^ Additionally, elevated 3-OHB and 2-OHB concentrations are associated with gestational diabetes, signaling elevated lipid metabolism under conditions of insulin resistance and glutathione synthesis to combat the associated oxidants.^47,48^ In this study, we adjusted for maternal diabetes, so associations of 3-OHB with 2-OHB, 2-aminobutyrate, pyroglutamate, and glutathione may be a sign of increased glutathione synthesis to manage increased oxidative metabolism.

The cord serum and placenta metabolic profiles were associated with risk of non-TD and may reflect altered fetal lipid metabolism. It has been reported that plasma 3-OHB concentrations measured from heel-stick samples collected from neonates are low in the first 12 hours after birth and peak between 48 and 72 hours.^49^ However, mean cord serum 3-OHB concentrations collected shortly after birth in our study were higher and positively associated with risk of non-TD. Non-TD is associated with various phenotypes, including attention-deficit/hyperactivity disorder (ADHD) and speech or other learning delays.^10^ In an umbilical cord serum lipidomic analysis, circulating acylcarnitines were positively associated with symptoms of both ASD and ADHD at age 2 years.^50^ There has been some work to suggest that higher plasma 3-OHB levels might correlate with decreased receptive language between 2 and 5 years in typically developing children.^51^ ADHD symptoms have been associated with broader changes lipid metabolism across multiple lipid classes.^50^ Thus, the placental and cord metabolic profiles reported here could be signs of altered cellular and lipid metabolism linked to impaired neurodevelopment that is detectable at birth.

### Strengths and Limitations

This analysis has several strengths, including the prospective birth cohort design and sample size to evaluate metabolism in the maternal-placental-fetal unit. The study design allows insight into a population at high familial risk for recurrent ASD.^52,53^ This study also has some limitations. Given the high heritability of ASD, these findings may not be generalizable to the wider population. The study design also means the possibility of residual confounding persists, although we adjusted for a variety of potential confounders. Here, we investigated the polar metabolome using nuclear magnetic resonance–based metabolomics, which allows for absolute quantitation (rather than relative abundance) of metabolites; however, the method can only measure those metabolites in the nanomole or micromole per liter range and greater. Future analyses using complementary approaches, including nonpolar lipidomics, will improve our understanding of perinatal metabolism and neurodevelopment. Furthermore, the non-TD group was relatively small, and this is reflected in wide CIs, particularly in the adjusted analyses. Future research is needed to corroborate these findings in similar high-risk cohorts, as well as in low-risk cohorts from the general population.

## Conclusions

In this cohort study of maternal, placenta, and fetal metabolism and neurodevelopment, we observed that the placental and umbilical cord metabolomes, which were both collected at delivery, were highly correlated and associated with risk of non-TD. Moreover, we observed that 3-OHB was an important metabolite in maternal third trimester serum and cord serum and, further, that lipid-based metabolism and oxidative stress were highly correlated in the placenta and cord serum. These findings suggest maternal and fetal lipid metabolisms are important in neurodevelopment.

## Notes

Supplement 1. **eFigure 1.** Diagram of Samples Included in This Analysis **eTable 1.** Covariates for Each Analysis and Each Model **eFigure 2.** The Directed Acyclic Graph for the Relationships Between the Maternal Third Trimester Serum Metabolome, Placental Metabolome, and Umbilical Cord Blood Metabolome **eFigure 3.** The Directed Acyclic Graph for the Relationships Between Neurodevelopmental Outcomes and 3-Hydroxybutyrate Levels in Maternal Third Trimester Serum, Placenta, and Umbilical Cord Serum **eFigure 4.** Spearman Correlations Between Maternal Third Trimester Serum Metabolites **eFigure 5.** Spearman Correlations Between Placental Metabolites **eFigure 6.** Spearman Correlations Between Umbilical Cord Serum Metabolites **eFigure 7.** Spearman Correlations Between Placental Metabolites and Maternal Third Trimester Serum Metabolites **eFigure 8.** Spearman Correlations Between Umbilical Cord Serum Metabolites and Maternal Third Trimester Serum Metabolites **eFigure 9.** Spearman Correlations Between Placental Metabolites and Umbilical Cord Serum Metabolites **eFigure 10.** Metabolite Loadings for the First Latent Placenta Variate and the First Latent Cord Blood Variate

Supplement 2. **eTable 2.** Estimates of the Relationship Between Each Maternal Third Trimester Serum Metabolite and Each Placental Metabolite **eTable 3.** Estimates of the Relationship Between Each Maternal Third Trimester Serum Metabolite and Each Umbilical Cord Serum Metabolite **eTable 4.** Estimates of the Relationship Between Each Placental Metabolite and Each Umbilical Cord Serum Metabolite

Supplement 3.
Data Sharing Statement
