# Supplementary material for: Neurodevelopment and Metabolism in the Maternal-Placental-Fetal Unit
Source: JAMA Netw Open. 2024 May 28;7(5):e2413399. doi: 10.1001/jamanetworkopen.2024.13399 (PMC11134213; doi:10.1001/jamanetworkopen.2024.13399)
Supplement: Supplement 1. — eFigure 1. Diagram of Samples Included in This Analysis eTable 1. Covariates for Each Analysis and Each Model eFigure 2. The Directed Acyclic Graph for the Relationships Between the Maternal Third Trimester Serum Metabolome, Placental Metabolome, and Umbilical Cord Blood Metabolome eFigure 3. The Directed Acyclic Graph for the Relationships Between Neurodevelopmental Outcomes and 3-Hydroxybutyrate Levels in Maternal Third Trimester Serum, Placenta, and Umbilical Cord Serum eFigure 4. Spearman Correlations Between Maternal Third Trimester Serum Metabolites eFigure 5. Spearman Correlations Between Placental Metabolites eFigure 6. Spearman Correlations Between Umbilical Cord Serum Metabolites eFigure 7. Spearman Correlations Between Placental Metabolites and Maternal Third Trimester Serum Metabolites eFigure 8. Spearman Correlations Between Umbilical Cord Serum Metabolites and Maternal Third Trimester Serum Metabolites eFigure 9. Spearman Correlations Between Placental Metabolites and Umbilical Cord Serum Metabolites eFigure 10. Metabolite Loadings for the First Latent Placenta Variate and the First Latent Cord Blood Variate [file jamanetwopen-e2413399-s001.pdf]

Supplemental Online Content

Parenti M, Schmidt RJ, Tancredi DJ, Hertz-Picciotto I, Walker CK, Slupsky CM. Neurodevelopment and metabolism in the maternal-placental-fetal unit. *JAMA Netw Open*. 2024;7(5):e2413399. doi:10.1001/jamanetworkopen.2024.13399

eFigure 1. Diagram of Samples Included in This Analysis.
eTable 1. Covariates for Each Analysis and Each Model
eFigure 2. The Directed Acyclic Graph for the Relationships Between the Maternal Third Trimester Serum Metabolome, Placental Metabolome, and Umbilical Cord Blood Metabolome.
eFigure 3. The Directed Acyclic Graph for the Relationships Between Neurodevelopmental Outcomes and 3-Hydroxybutyrate Levels in Maternal Third Trimester Serum, Placenta, and Umbilical Cord Serum.
eFigure 4. Spearman Correlations Between Maternal Third Trimester Serum Metabolites
eFigure 5. Spearman Correlations Between Placental Metabolites
eFigure 6. Spearman Correlations Between Umbilical Cord Serum Metabolites
eFigure 7. Spearman Correlations Between Placental Metabolites and Maternal Third Trimester Serum Metabolites
eFigure 8. Spearman Correlations Between Umbilical Cord Serum Metabolites and Maternal Third Trimester Serum Metabolites
eFigure 9. Spearman Correlations Between Placental Metabolites and Umbilical Cord Serum Metabolites
eFigure 10. Metabolite Loadings for the First Latent Placenta Variate and the First Latent Cord Blood Variate

This supplemental material has been provided by the authors to give readers additional information about their work.

© 2024 Parenti M et al. *JAMA Network Open.*

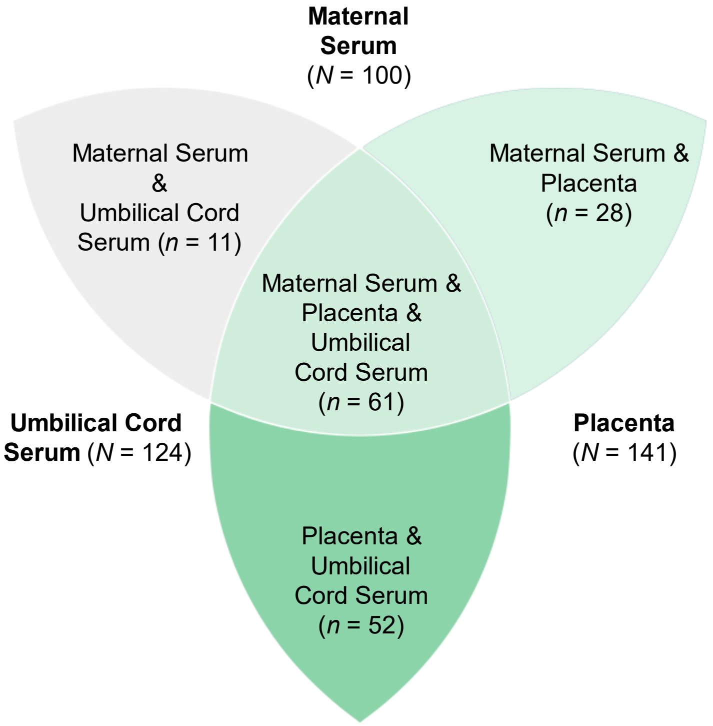

eFigure 1. Diagram of samples included in this analysis.

There were a total of 100 maternal serum samples, 141 placental samples, and 124 umbilical cord serum samples. Of these, 89 mother–infant dyads provided at least a maternal serum sample and a placental sample, 72 mother–infant dyads provided at least a maternal serum sample and an umbilical cord serum sample, and 113 mother–infant dyads provided at least a placental sample and an umbilical cord sample. In total, 61 mother–infant dyads provided a maternal serum sample, placental sample, and umbilical cord serum sample.

© 2024 Parenti M et al. *JAMA Network Open.*

eTable 1. Covariates for each analysis and each model

Analysis	Model 1 [a]	Model 2 [b]
Maternal Serum–Placenta	Birth year, gestational age at maternal serum sample collection, fasted time at serum sample collection, and gestational age at delivery	Birth year, gestational age at maternal serum collection, gestational age at delivery, fetal sex, fasted time at serum sample collection, home ownership, maternal education, maternal race and ethnicity, and maternal metabolic condition
Maternal Serum–Cord Serum	Birth year, gestational age at maternal serum sample collection, fasted time at serum sample collection, and gestational age at delivery	Birth year, gestational age at maternal serum collection, gestational age at delivery, fetal sex, fasted time at maternal serum sample collection, home ownership, maternal education, maternal race and ethnicity, and maternal metabolic condition
Placenta–Cord Serum	Birth year and gestational age at delivery	Birth year, gestational age at delivery, fetal sex, delivery mode, home ownership, maternal education, maternal race and ethnicity, maternal metabolic conditions, and prenatal vitamin use in the first month of pregnancy

[a] Model 1 was minimally adjusted model adjusted for variables related to sample collection.
[b] Model 2, a fully adjusted model with variables selected *a priori* using a directed acyclic graph.

© 2024 Parenti M et al. *JAMA Network Open.*

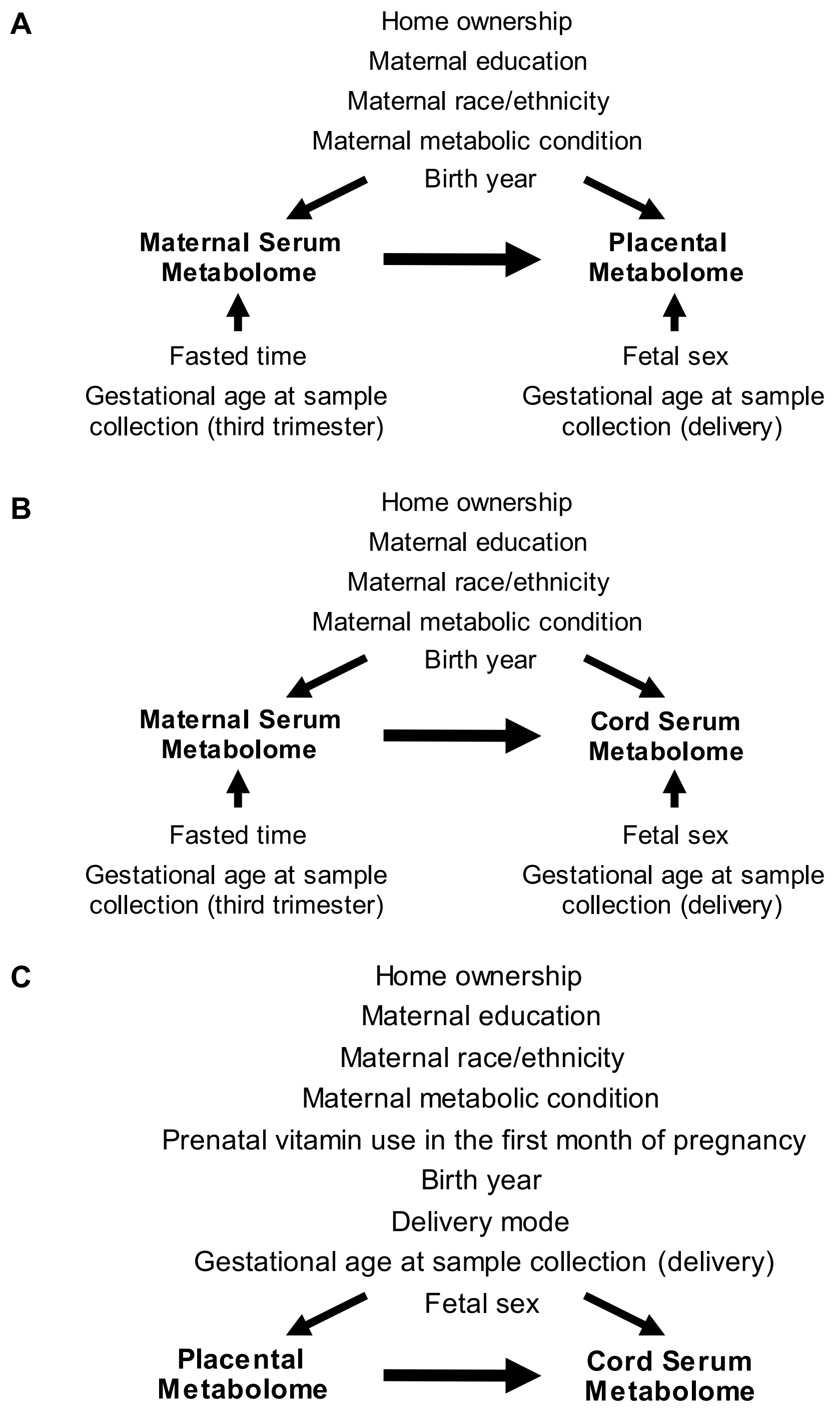

eFigure 2. The directed acyclic graphs for the relationships between the maternal third trimester serum metabolome, placental metabolome, and umbilical cord blood metabolome.

We present here simplified directed acyclic graphs identifying confounders and precision variables for each metabolome. Maternal race and ethnicity, education, and home ownership were included as proxies of social and economic inequities. Maternal metabolic condition considers pre-pregnancy BMI, hypertensive disorders during pregnancy, and diabetes during pregnancy.

© 2024 Parenti M et al. *JAMA Network Open.*

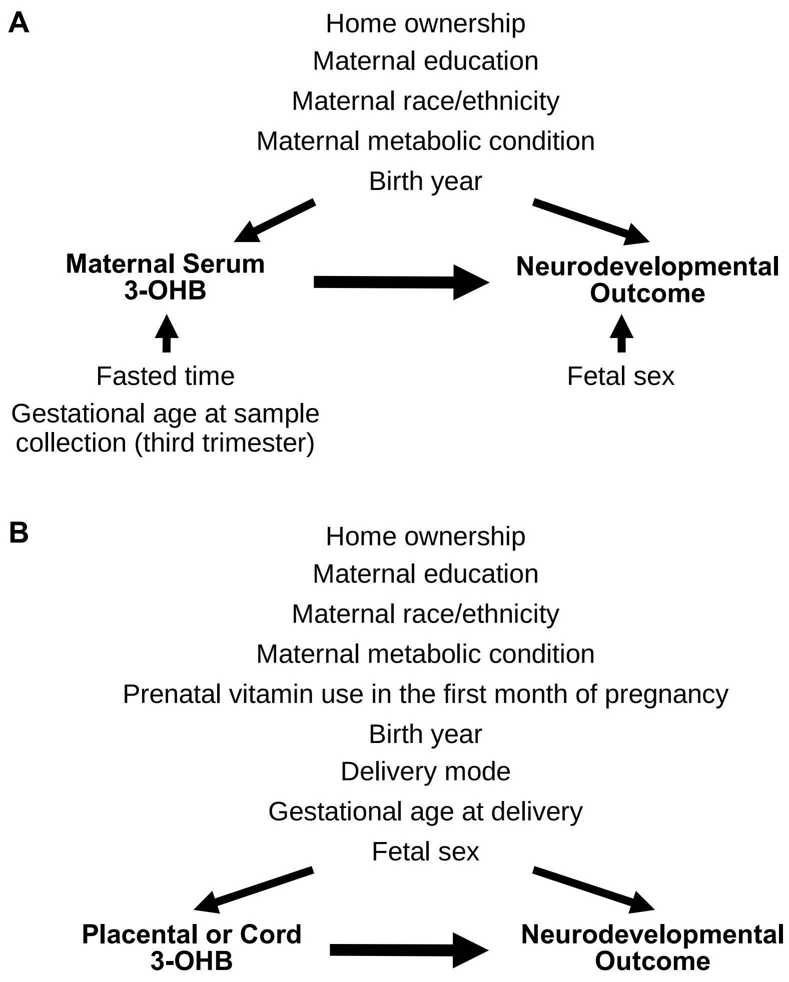

eFigure 3. The directed acyclic graphs for the relationships between neurodevelopmental outcomes and 3-hydroxybutyrate levels in maternal third trimester serum, placenta, and umbilical cord serum.

We present here simplified directed acyclic graphs identifying confounders and precision variables. Maternal race and ethnicity, education, and home ownership were included as proxies of social and economic inequities. Maternal metabolic condition considers pre-pregnancy BMI, hypertensive disorders during pregnancy, and diabetes during pregnancy.

© 2024 Parenti M et al. *JAMA Network Open.*

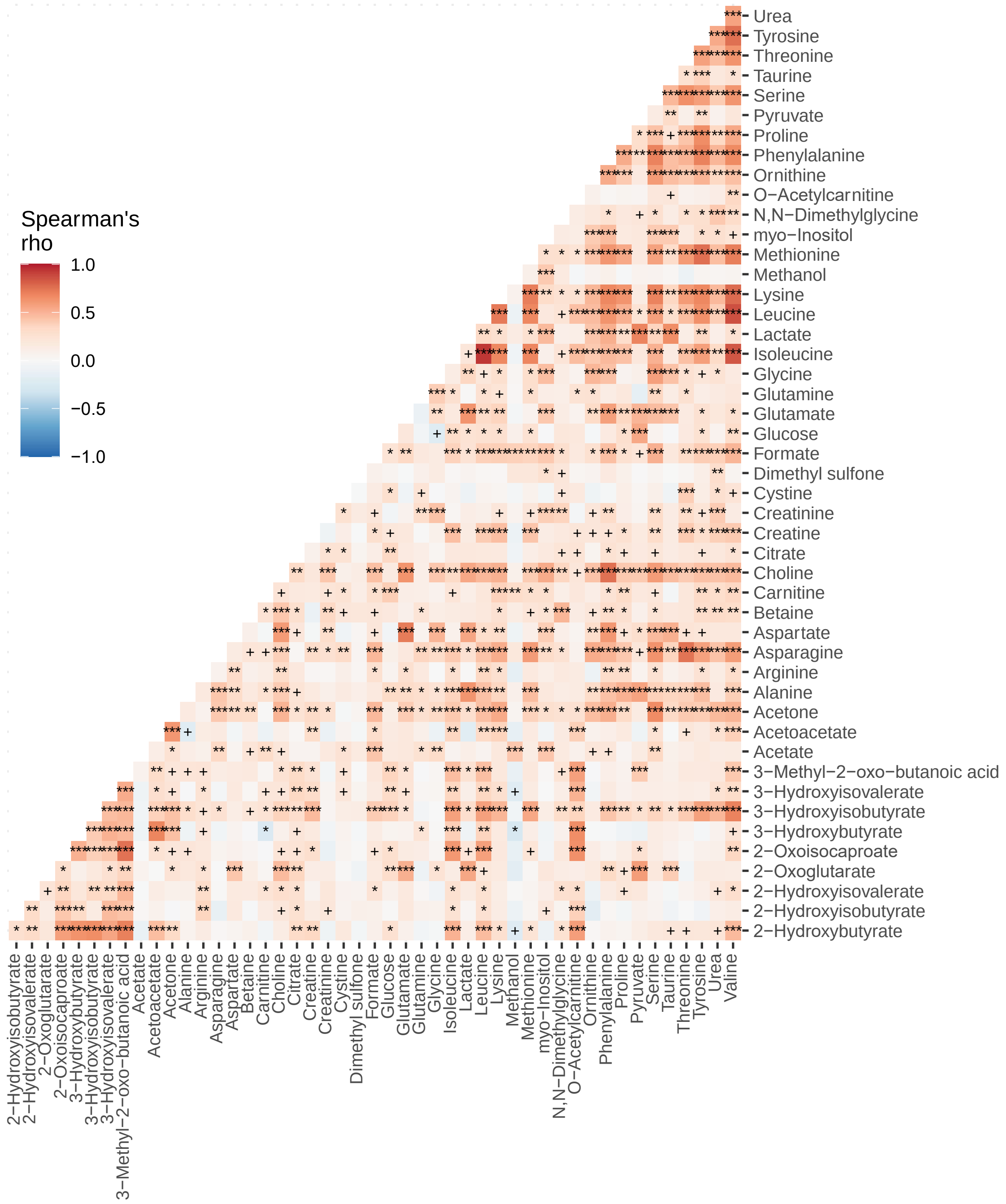

eFigure 4. Spearman correlations between maternal third trimester serum metabolites (*n* = 100).

Metabolites that remained significant after false discovery rate correction are marked with stars. For $q < 0.001$, ***; $q < 0.01$, **; $q < 0.05$, *; and $q < 0.1$, +.

© 2024 Parenti M et al. *JAMA Network Open.*

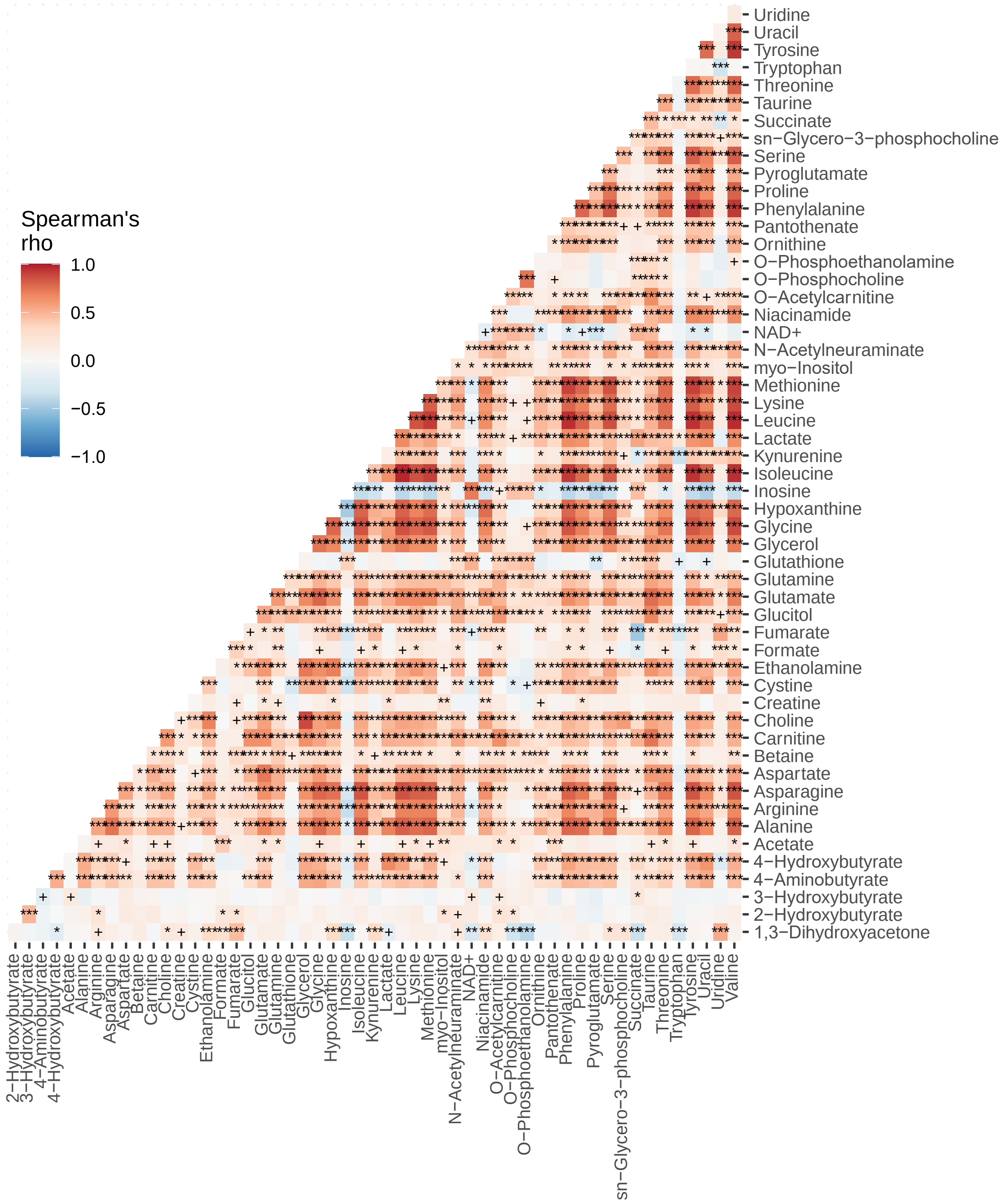

eFigure 5. Spearman correlations between placental metabolites (*n* = 141).
Metabolites that remained significant after false discovery rate correction are marked with stars. For *q* < 0.001, ***; *q* < 0.01, **; *q* < 0.05, *; and *q* < 0.1, +.

© 2024 Parenti M et al. *JAMA Network Open.*

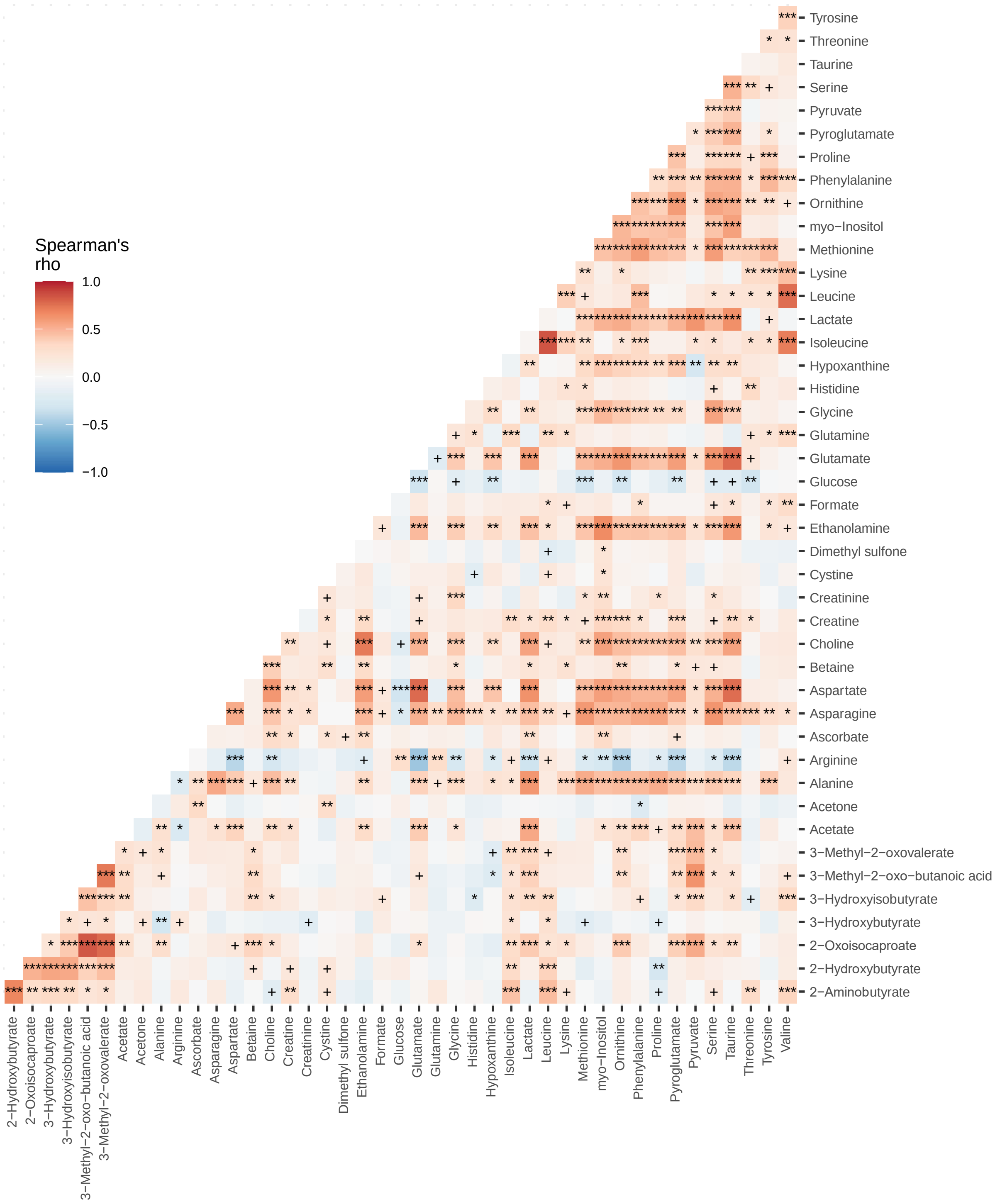

eFigure 6. Spearman correlations between umbilical cord serum metabolites (*n* = 124).

Metabolites that remained significant after false discovery rate correction are marked with stars. For $q < 0.001$, ***; $q < 0.01$, **; $q < 0.05$, *; and $q < 0.1$, +.

© 2024 Parenti M et al. *JAMA Network Open.*

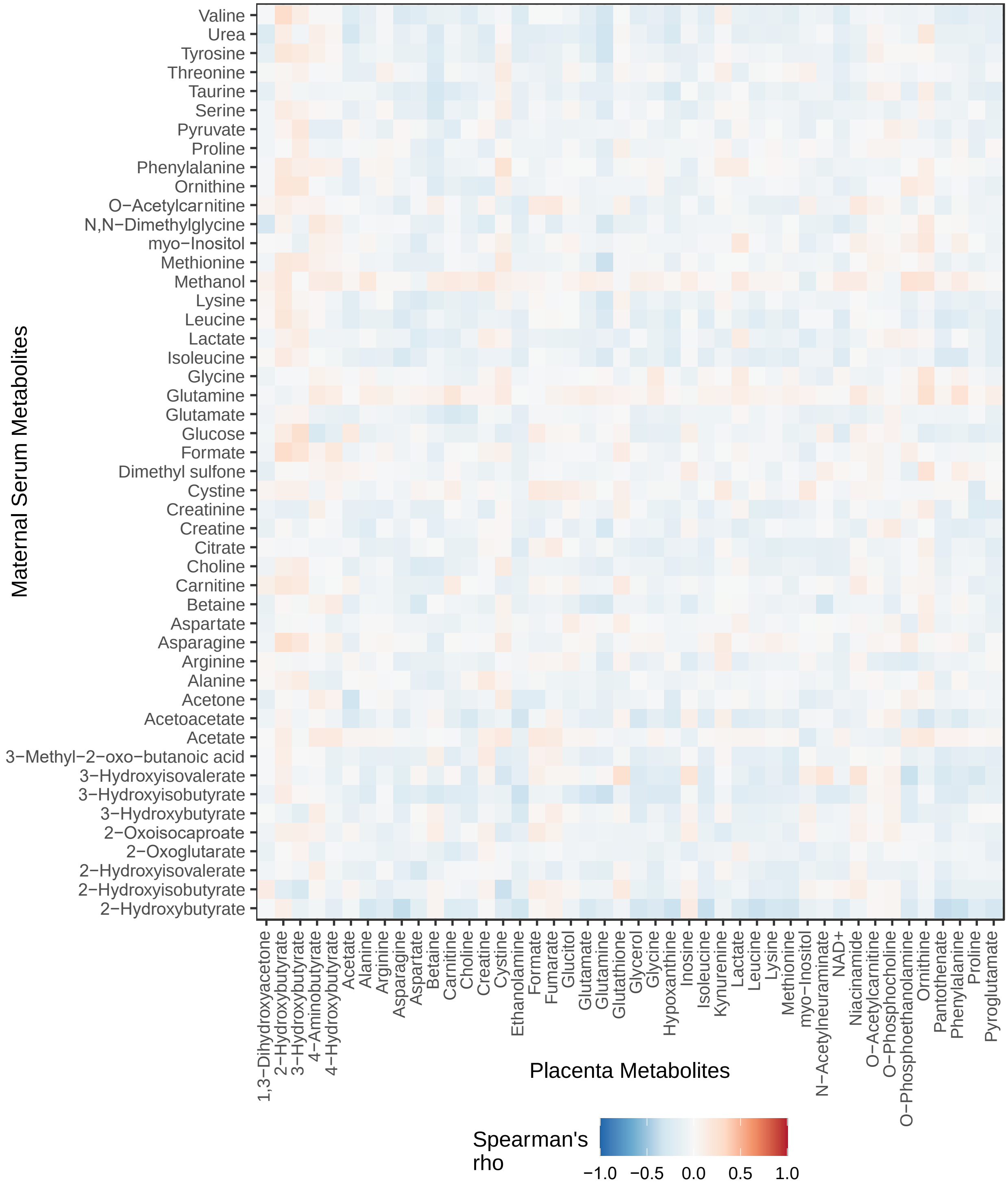

eFigure 7. Spearman correlations between placental metabolites and maternal third trimester serum metabolites.

Metabolites that remained significant after false discovery rate correction are marked with stars. For *q* < 0.001, ***; *q* < 0.01, **; *q* < 0.05, *; and *q* < 0.1, +.

© 2024 Parenti M et al. *JAMA Network Open.*

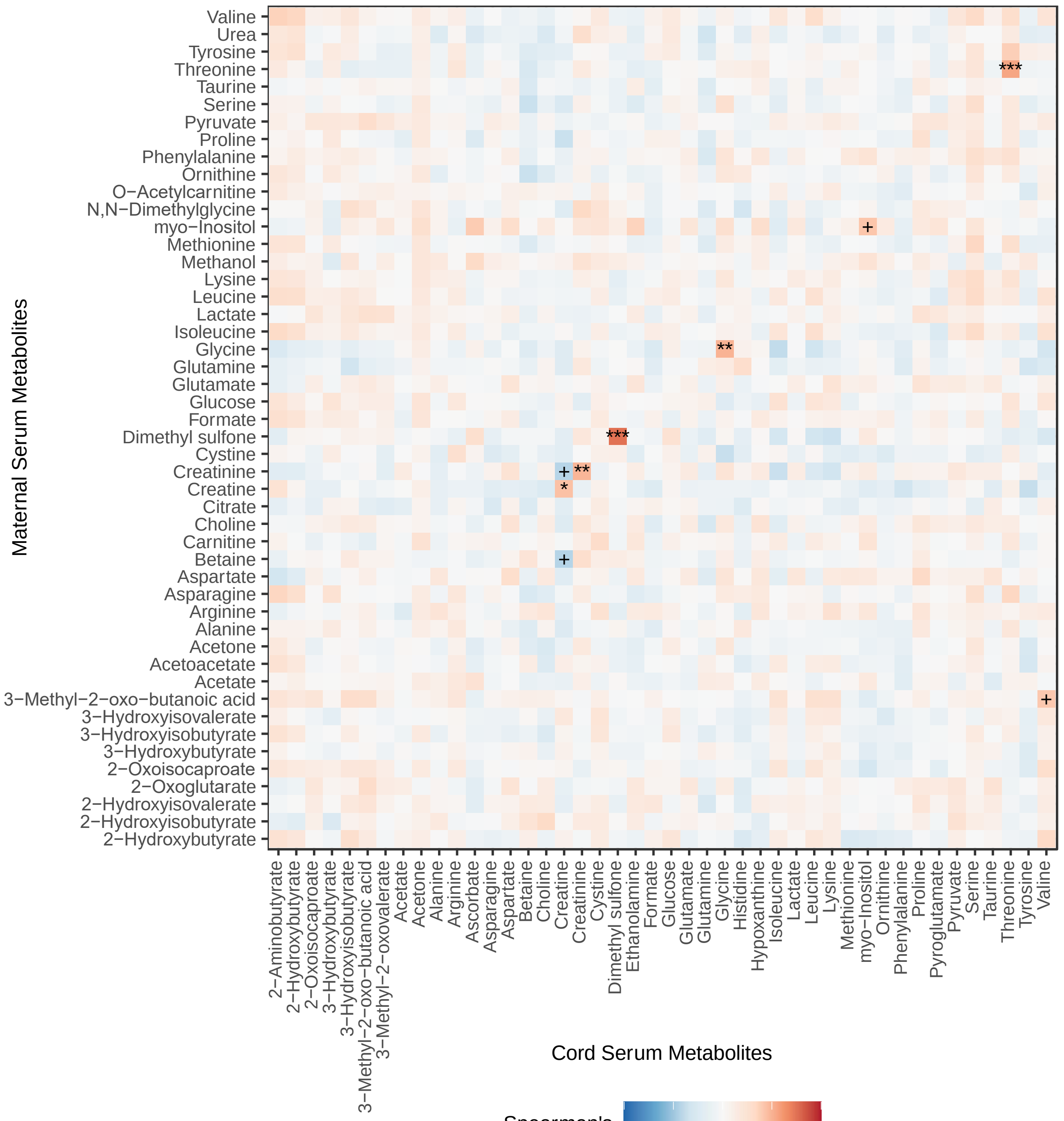

eFigure 8. Spearman correlations between umbilical cord serum metabolites and maternal third trimester serum metabolites.

Metabolites that remained significant after false discovery rate correction are marked with stars. For $q < 0.001$, ***; $q < 0.01$, **; $q < 0.05$, *; and $q < 0.1$, +.

© 2024 Parenti M et al. *JAMA Network Open.*

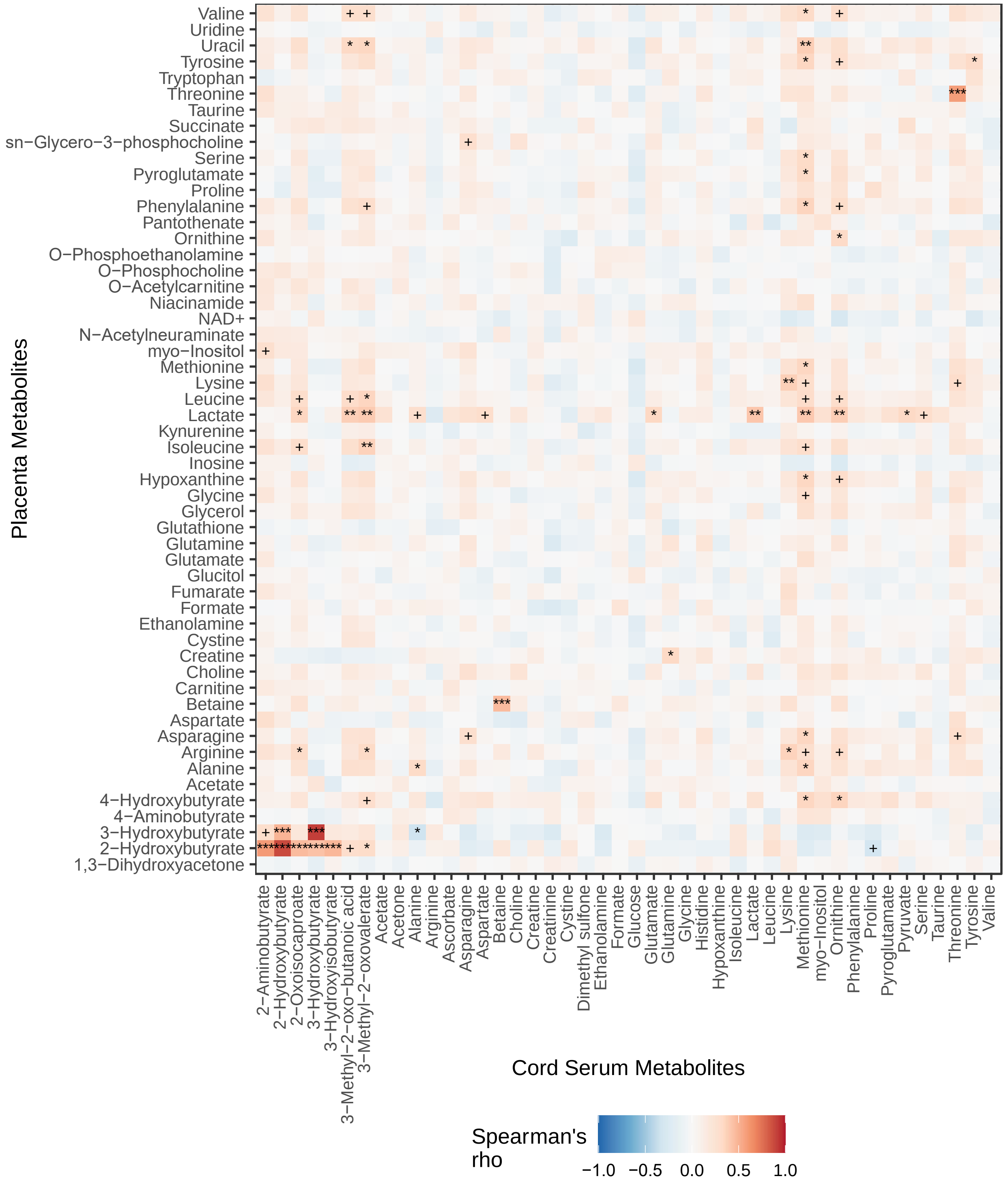

eFigure 9. Spearman correlations between placental metabolites and umbilical cord serum metabolites.

Metabolites that remained significant after false discovery rate correction are marked with stars. For $q < 0.001$, ***; $q < 0.01$, **; $q < 0.05$, *; and $q < 0.1$, +.

© 2024 Parenti M et al. *JAMA Network Open.*

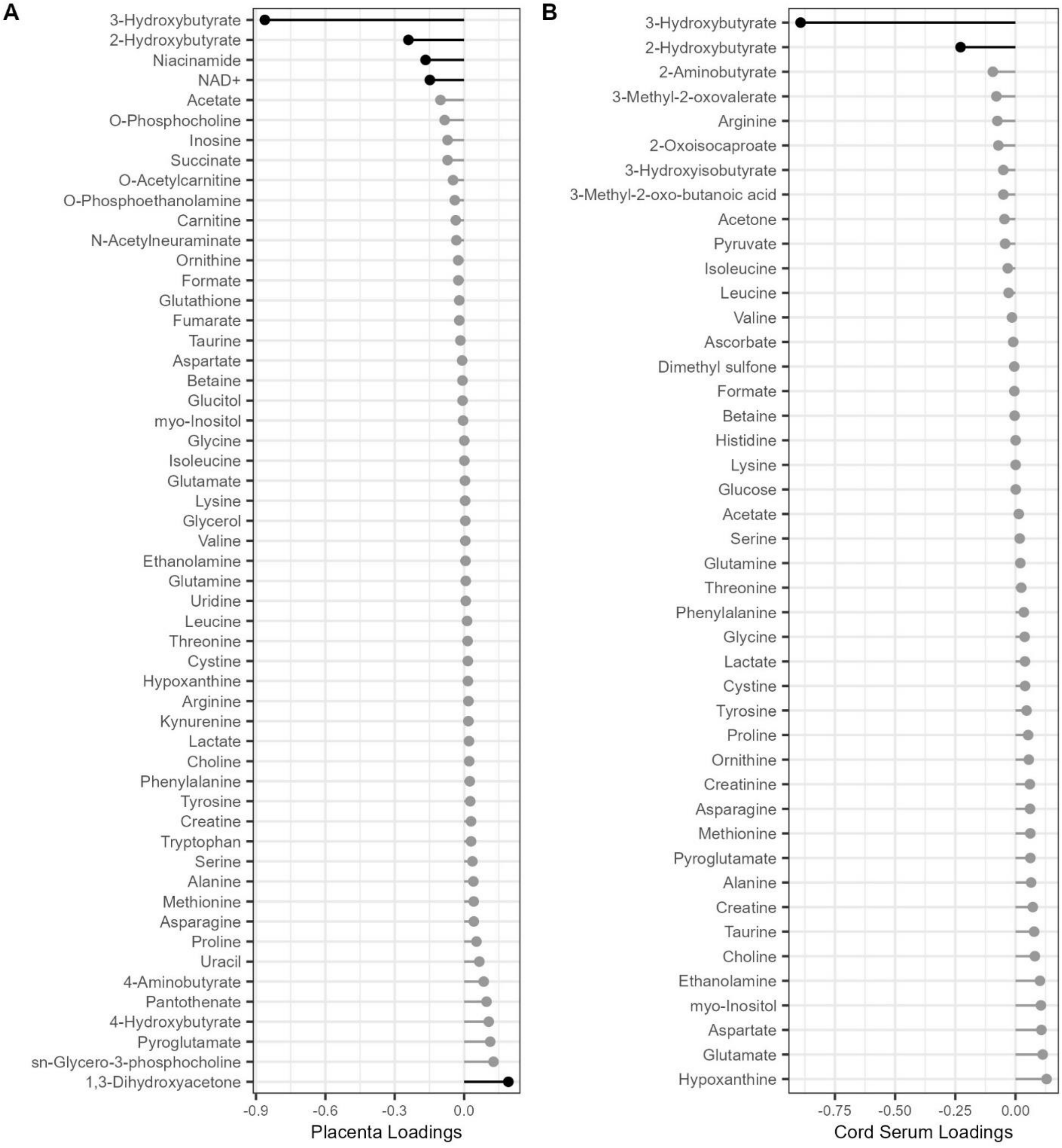

eFigure 10. Metabolite loadings for the (A) first latent placenta variate and (B) the first latent cord blood variate.

Loadings are projected so that multiplying the loading by the original concentration (adjusted for Model 2 covariates) results in the latent variate score. Since the sum of squares of the metabolite loadings for a variate is equal to 1, placenta metabolites whose squared loading were greater than 1/54 and cord serum metabolites whose squared loading were greater than 1/44 contributed more than average to their respective variate and thus could be considered important. These metabolites are presented in black, and all other metabolites are presented in grey.

© 2024 Parenti M et al. *JAMA Network Open.*
