# Supplementary material for: Neurodevelopment and Metabolism in the Maternal-Placental-Fetal Unit
Source: JAMA Netw Open. 2024 May 28;7(5):e2413399. doi: 10.1001/jamanetworkopen.2024.13399 (PMC11134213; doi:10.1001/jamanetworkopen.2024.13399)
Supplement: Supplement 3. — Data Sharing Statement [file jamanetwopen-e2413399-s003.pdf]

# Data Sharing Statement

Parenti. Neurodevelopment and Metabolism in the Maternal-Placental-Fetal Unit. *JAMA Netw Open*. Published May 28, 2024. doi:10.1001/jamanetworkopen.2024.13399

**Data**
**Data available:** No

**Additional Information**
**Explanation for why data not available:** We will provide data under reasonable request; however, we have elected to not make it publicaly available.
